# Neural Activity while Imitating Emotional Faces is Related to Both Lower and Higher-Level Social Cognitive Performance

**DOI:** 10.1038/s41598-017-01316-z

**Published:** 2017-04-28

**Authors:** Colin Hawco, Natasa Kovacevic, Anil K. Malhotra, Robert W. Buchanan, Joseph D. Viviano, Marco Iacoboni, Anthony R. McIntosh, Aristotle N. Voineskos

**Affiliations:** 1 Campbell Family Mental Health Institute, Centre for Addiction and Mental Health, Department of Psychiatry, University of Toronto, 250 College St., Toronto, M5T 1R8 ON Canada; 2 Rotman Research Institute, Baycrest Geriatric Centre, 3560 Bathurst Street, Toronto, M6A 2E1 Ontario Canada; 3 Zucker Hillside Hospital, 75-59 263rd St, Glen Oaks, 11004 NY United States; 4 Maryland Psychiatric Research Center, 55 Wade Ave, Catonsville, 21228 MD United States; 5 University of California – Los Angeles, Los Angeles, 90095 CA United States

## Abstract

Imitation and observation of actions and facial emotional expressions activates the human fronto-parietal mirror network. There is skepticism regarding the role of this low-level network in more complex high-level social behaviour. We sought to test whether neural activation during an observation/imitation task was related to both lower and higher level social cognition. We employed an established observe/imitate task of emotional faces during functional MRI in 28 healthy adults, with final analyses based on 20 individuals following extensive quality control. Partial least squares (PLS) identified patterns of relationships between spatial activation and a battery of objective out-of-scanner assessments that index lower and higher-level social cognitive performance, including the Penn emotion recognition task, reading the mind in the eyes, the awareness of social inference test (TASIT) parts 1, 2, and 3, and the relationships across domains (RAD) test. Strikingly, activity in limbic, right inferior frontal, and inferior parietal areas during imitation of emotional faces correlated with performance on emotion evaluation (TASIT1), social inference - minimal (TASIT2), social inference - enriched (TASIT3), and the RAD tests. These results show a role for this network in both lower-level and higher-level social cognitive processes which are collectively critical for social functioning in everyday life.

## Introduction

Social interactions are complex processes which are built upon the perception and understanding of the actions, intentions, and mental state of others. Social cognitive neuroscience has suggested a dichotomy between low-level processes such as understanding motor actions or emotional processing and higher-level processes such as inferring the mental states of others (e.g. theory of mind) or detecting deception and sarcasm. This apparent dichotomy between lower level and higher level social cognitive processes is supported by behavioral^1^, neuroimaging^2^ and lesion studies^3, 4^. Neuroimaging studies have primarily examined these processes with two approaches: using tasks relevant to the perception of and interaction with other people’s actions (action observation and imitation), and using tasks relevant to infer the beliefs and desires of the other person (mentalizing). Lower level social cognitive processes such as action interpretation have been associated with activation of a lateral frontal-parietal network, the putative human analogue to the mirror neuron system observed in monkeys^2, 5^. Higher-level social cognitive processes, such as theory of mind, have been associated with activation of cortical midline regions, including the medial prefrontal cortex, posterior cingulate cortex, and precuneus, as well as temporoparietal junction and temporal pole. This network is also known as the ‘mentalizing’ system. Dual processing models have been proposed in which the lateral fronto-parietal network supports relatively automatic processes such as identifying motor actions while the mentalizing network supports controlled processes of attributing actions to complex social causes^6–8^.

Most neuroimaging studies examining the lateral fronto-parietal network have made use of hand actions or other forms of simple motor stimuli^9^. As such, the role of this system has been mainly considered in the context of perceiving gross motor actions. Some have taken the concept of mirroring one step further, by arguing that the lateral fronto-parietal network is important for emotional empathy by permitting humans to feel what others feel, and potentially to assess or interpret emotional cues and social behaviour^10–12^. Investigating this issue may be best served by paradigms utilizing socially relevant stimuli as opposed to simple motor acts. One such paradigm is the imitation and observation of emotional faces, which has been shown to activate the lateral fronto-parietal ‘mirror’ network^13–15^. This is consistent with the notion that activation of this network can play a role in allowing people to empathize by imitating emotions^11, 12^. Activity in the right IFG has been positively correlated with self-report measures of empathy in healthy children^15^, and has been shown to be more active in normally developing children than those with autism^13^, with activity in autistic children inversely correlated with social impairment. However, the IFG has also been implicated in some higher level social cognitive processes, specifically the inhibiting self-perspective in social judgements^16, 17^. Recent work has also shown structural changes in right fronto-parietal cortex in more socially impaired people with schizophrenia^18, 19^, a group of individuals in whom higher-level social cognitive processes such as theory of mind are more prominently affected^20^. These findings raise the question of whether neural activation during basic mirroring tasks is related to higher-level social cognitive abilities.

Meta-analysis of fMRI data has shown little overlap between activity in the brain regions associated with the mirroring and mentalizing systems across a range of tasks^2^, and brain lesion studies have suggested a dissociation between mirroring and mentalizing regions^3, 4^. As such, mirroring and mentalizing were initially thought to represent dichotomous systems which function relatively independently, and the role of mirroring in higher-level social cognition is a contentious issue^21–23^. However, there is a growing body of evidence that the mentalizing and mirroring systems may interact during more complex social cognitive processing, such as watching social videos^24–27^. Co-activation of mentalizing and mirroring regions has also been noted when viewing agents performing irrational actions, suggesting aspects of the mentalizing system may facilitate interpreting unexpected actions^28–30^. The mirroring and mentalizing system may work together during social cognitive processing, although a specific role of the mirror system in higher-level cognitive processing has yet to be established.

The purpose of this study is to determine whether brain regions activated during a facial emotion mirroring task are related to lower-level social cognitive performance, higher-level social cognitive performance, or both. We utilized a process specific social mirroring task (the facial imitate/observe task^13–15^) inside the fMRI, along with a battery of objective out-of-scanner social tests ranging from basic emotional-perception tasks to complex higher level social cognitive tasks such as detection of lies or sarcasm. Brain-behavior relationships were examined using the spatio-temporal partial least squares (PLS), a non-parametric multivariate approach^31, 32^. PLS identifies latent variables (LVs), linear combinations of fMRI signal amplitude at each voxel across time and a design matrix which can consist of either experimental conditions or correlations across conditions with behavioral scores. Each LV expresses a pattern of the common covariance between the design and the fMRI data. PLS is designed to handle data with high collinearity while simultaneously capturing essential non-redundant relationships among the data^31^, overcoming the confounding influence of collinear variables in traditional multiple regression^33^. This makes PLS an excellent approach to uncover relationships between brain activity and a set of social cognitive scores which are likely highly interrelated. We hypothesized that a) consistent with previous studies, imitating emotional faces would activate the mirroring network, more prominently in the imitate than the observe condition, and b) neural activity during imitation of emotional faces specifically (rather than imitating neutral faces or observing faces) would be associated with both lower-level and higher-level social cognitive performance.

## Results

### Task Effects

**Figure 1:**
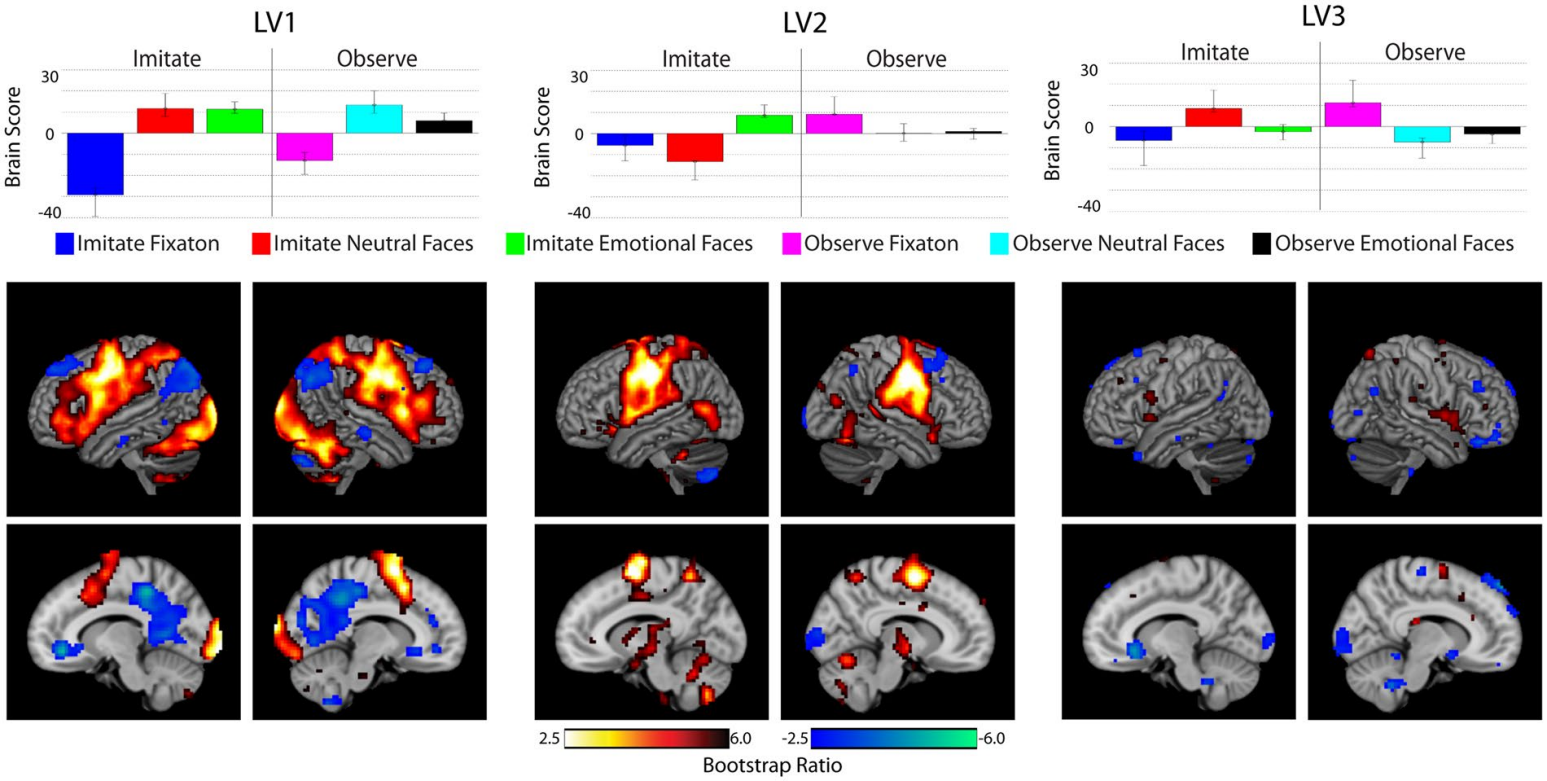
Results from the first latent variable using task PLS analysis. Data is shown for LV1, 2 and 3. The top panel shows the design pattern (the contribution of each condition to the LV). Error bars are 95% confidence interval derived from the bootstrap analysis. The bottom panel shows the voxel pattern from the first lag of the PLS analysis rendered on the cortex. Sagittal slices are shown for X = −10 (left) and X = 10 (right). Voxel intensity is displayed as bootstrap ratio, a measure of reliability of voxels within the LV. Red-yellow regions show the pattern described by the design pattern, while blue regions show the opposite pattern.

Task PLS is a multivariate model-free approach to identify spatial patterns which share relationships across the experimental conditions. A task PLS was run on the data to replicate previous task-based analysis, and examine the underlying pattern of task task-evoked spatial activity. PLS generated 6 LVs (as there were three trial types, emotional faces, neutral faces, and fixation, and two conditions, Imitate and Observe). Permutation analysis (see methods below) showed significance (p < 0.05) in the first three LVs, which accounted for 61.6%, and 14.5%, and 12.9% of crossblock covariance respectively (Fig. 1). LV 1 was related to viewing faces as opposed to fixation trials, and showed a pattern related to the lateral frontal-parietal network similar to the results of previous studies^13–15^. LV 1 was related to viewing faces in general rather than distinguishing between emotional and neutral faces, demonstrating that this pattern of faces >fixation in the mirroring network is the dominant pattern of neural activity within this task analysis. LVs 2 and 3 explain some of the remaining variance in the data not accounted for the pattern presented in LV 1, representing interaction effects showing different patterns of activity across the Imitate and Observe conditions. LV 2 shows a large amount of overlap with LV 1, suggesting some of the variance within those regions is explained by aspects of both LVs 1 and 2. The majority of the variance explained by LV 1, but some additional variance within those voxels is explained by the pattern of emotional faces >neutral and fixation in Imitate and increased activity in fixation trials for Observe, as seen in LV 2. LV 3 represents an interaction in which the relative effects of neutral faces and fixation are different in the Imitate and Observe tasks.

For consistency and replication of previous studies, we also ran a general linear model (GLM) in SPM8. The GLM showed a similar pattern as in previous studies and LV1 of the task PLS (see supplementary methods and Supplementary Figure 1).

### Behavioral PLS

**Figure 2:**
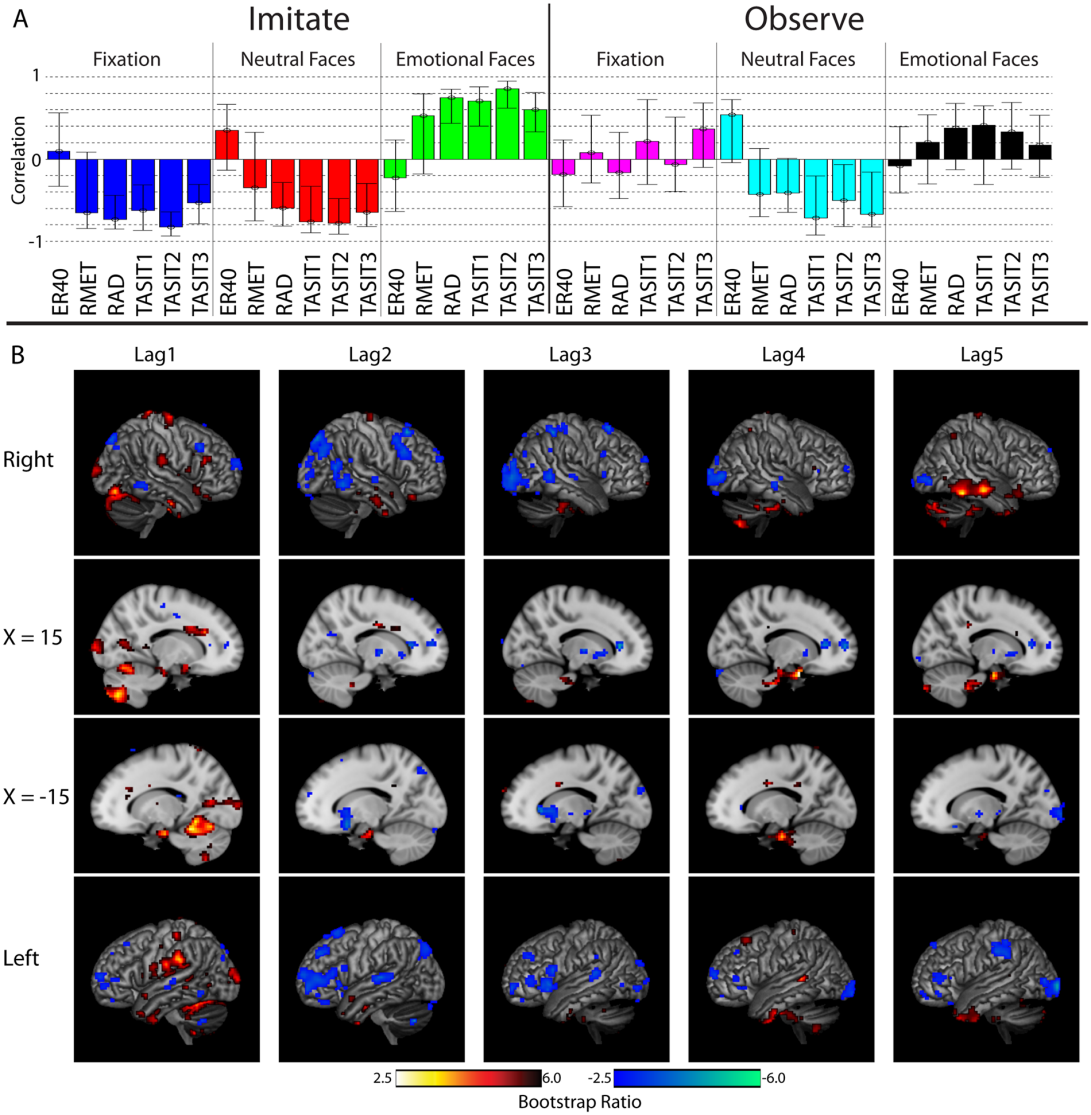
LV 2 of the behavioral PLS results. (**A**) Design pattern showing the pattern of correlations between brain signal for each during event type and each behavioral score within the voxel pattern. Error bars represent 95% confidence interval based off a bootstrapping analysis of 1000 iterations. (**B**) Voxel patterns for each lag, displayed as bootstrap ratio on the MNI152 brain. Data is shown for two sagittal views as well as rendered onto the cortex (8 mm search depth) to better visualize cortical activity. Red-yellow shows a correlation pattern matching the design pattern (**A**), while blue regions show the opposite pattern. Each lag represents a time point (TR) following stimuli onset (which occurred during ‘Lag0’).

Behavioral PLS examined the relationships between patterns of activity in the brain and social cognitive test scores. Scores from six social cognitive tasks were included: the Emotion Recognition (ER40)^34^, the Reading the Mind in the Eyes Test, RMET^35^, the Relationships Across Domains test (RAD)^36^, and the three subtests of the Awareness of Social Inference Test Revised (TASIT-R)^37^. TASIT1 can be considered a test of lower-level social cognitive functioning, while TASIT2 and TASIT3 are high-level tests. The behavioral PLS generated 36 LVs, as there were six experimental conditions (emotional faces, neutral faces, and fixation, separately for Imitate and Observe, and six social cognitive tests). Overall permutation analysis showed significance in the first two LVs, which accounted for 23% and 13.2% of crossblock covariance respectively. However, the split-half permutation reliability analysis (see methods) demonstrated that the relationship between the design pattern and brain pattern was not reliable within LV 1 and as such it was not considered further. In LV 2, neural activity during imitation of emotional faces was positively correlated with RAD, TASIT1, TASIT2, and TASIT3 (Fig. 2, top panel). Neural activity for Fixation and Neutral faces during Imitate, as well as Neutral faces during Observe, was negatively correlated with those same test scores. As such, LV 2 shows a pattern of correlations during the imitation of emotional faces related to performance on TASIT1, TASIT2, TASIT3, and RAD.

The pattern of brain activity in LV 2 (Fig. 2, bottom panel) when imitating emotional faces in lag 1 showed a positive relationship with performance on TASIT1 TASIT2, TASIT3, and RAD. Activity was present in right pars opercularis (within inferior frontal gyrus), right and left supramarginal gyrus, right motor cortex, bilateral anterior temporal regions, fusiform gyrus, and parahippocampal cortex. Activity was also noted in right anterior/mid cingulate, right rolandic operculum, right putamen, and the cerebellum. Activity in lags 4 and 5, which positively correlated with social cognitive performance, was notably present in anterior temporal regions as well as in posterior middle and inferior right temporal gyri. Several regions showed negative relationships to the design pattern (thus greater fMRI activity while imitating emotional faces in individuals with poorer social cognitive performance), including the left inferior frontal gyrus (pars operculum and pars triangularis), and bilaterally in the basal ganglia (greatest in left putamen and right globus pallidus).

## Discussion

The behavioral PLS results of this study showed a robust relationship between neural activity during a process-specific imitate-observe task and both lower-level and higher-level social cognitive performance. While a growing number of studies using complex stimuli have noted co-activation of mirroring and mentalizing regions^24, 27, 38^, co-activity cannot be taken to conclude that mirroring has a strong role in higher level social cognition. The novelty of our study is that we showed complex relationships of neural activity during a simple and process-specific mirroring task with performance on objective out-of-scanner assessments of higher-level social cognitive performance. In addition, the PLS approach allowed us to include several assessments in a single analysis, providing data driven evidence of which assessments best capture these brain-behaviour relationships. These behavioral relationships were specific to imitation of emotional faces, rather than neutral faces. Several regions outside the right fronto-parietal circuit, including left IFG and some mentalizing regions (e.g. TPJ), also showed negative correlations with social cognitive performance, suggesting either compensatory responses or over-activation in participants with poorer social cognition.

These findings of a relationship between activity in a simple mirroring task and higher level social cognitive processing scores suggest a relationship for the mirror network in higher-order social cognitive processing including complex tasks. Effective mentalizing in humans although likely primarily reliant on classic mentalizing regions may be further subserved by mirroring regions^39, 40^. Given that the relationships elicited were noted in assessments involving interpretation of dynamic interactive video tasks including human actors, it may be that such relationships are only evoked when we consider social cognition as a process involving integrating information from multiple processing levels. The mirroring network may not be solely for understanding the intentions and physical goals of motor acts^41, 42^. Our findings add to the debate regarding the role of this network in social cognition^2, 21, 22, 43, 44^. Several authors have argued that this network is important not only for imitation or action perception, but also for emotional empathy and possibly even in cognitive empathy (sometimes used interchangeably with theory of mind)^23, 42^, consistent with the theory of embodied simulation^10, 44^, despite debate to the contrary^21, 22, 43^. Through the combination of a process-specific in-scanner task, more naturalistic and objective social cognitive tests, and the PLS multivariate framework, we were able to uncover essential brain-behavior relationships for which traditional task contrasts or linear regression analyses may be less sensitive.

Higher level social cognitive processing involves concepts related to both theory of mind and cognitive empathy, both complex constructs which likely encompass several mental processes. To date, there is only limited evidence of overlap in neural activity between high-level tasks related to the mentalizing network and low-level tasks related to the mirroring network^2, 21, 22, 43, 45, 46^. However, task based analysis is often performed by contrasting conditions, and many mirroring tasks have utilized stimuli with little or no direct social relevance, or tasks designed to evaluate highly specific social cognitive processes which may not be well suited to capturing overlap within these systems^47^. By moving away from an ‘overlapping activity’ perspective into an approach focused on brain-behavior relationships, we were able to demonstrate that activity in the mirroring network is correlated with higher-level social cognitive abilities. This combination of pairing a process-specific task paradigm with dynamic social cognitive measures may be a powerful approach for probing relationships between social cognitive networks and real-life social cognitive abilities.

The behavioral PLS LV correlating neural activation during imitation of emotional faces with TASIT and RAD scores also included some regions which are often considered part of the mentalizing network. While these regions did not robustly activate during the task PLS, their presence in the behavioural PLS reflects activation during the imitate task that is relevant for social cognitive performance. Positive relationships with social cognition and emotional faces were observed in the temporal pole bilaterally, a region implicated in mentalizing and theory of mind^48^ as well as in processing deception^49^. Activity was also noted in the posterior region of the superior temporal sulcus, which may be of particular interest as this area has been implicated in both the mirroring/imitation network^50^ and the mentalizing network^48^. The superior temporal sulcus may serve as an integrative region, sharing information between these two networks^51^ and/or as serving as a relay point for high level visual information^5, 52^. The superior temporal sulcus has also been implicated in analyzing dynamic facial features^53, 54^, perceiving biological motion^55, 56^, and gaze perception^2^. As such, this region is involved with perceptual processes necessary for both low-level and high-level social cognition. It remains to be seen if this region may serve as an integrative hub between the mentalizing and mirroring networks, though the posterior superior temporal sulcus shares connectivity within both networks^51^ and has been implicated as an important region in social cognitive impairment in autism^57, 58^.

Studies showing increased connectivity between the right IFG (a critical hub of the mirroring network) and the mentalizing network provide evidence for functional communications between these networks^24, 59–62^. This connectivity between mirroring and mentalizing regions has been shown to be disrupted in autism^59, 63^, raising the possibility that disrupted social cognition in autism may be driven not only by localized effects within one of these systems but also as a network level disruption in how these systems interact. Likewise, social cognitive deficits are increasingly recognized as a core feature of schizophrenia, strongly related to overall functional outcomes^1^. People with schizophrenia have been noted to show reduced right IFG activity when imitating emotional faces as well as opposite relationships between self-reported empathy and right IFG activity compared to controls^64^. As social cognitive impairment is present at, or potentially preceding the onset of psychosis^20^, it is possible this relatively brief imitate/observe in-scanner task may be useful as a potential early risk marker of the disease.

We found that increased activity in the left IFG was associated with poorer social cognitive performance, as well as in clusters in the left medial frontal cortex and the TPJ^4, 46–48^. One study examining resting state network connectivity noted over-connectivity of the left IFG in the mirroring network in participants with autism, consistent with our finding that over activity in the left IFG is associated with poorer social cognitive performance^65^. A number of regions outside the mirroring or mentalizing networks also showed a negative relationship with social cognitive performance, including the basal ganglia, thalamus, occipital cortex and superior colliculus. These regions may be involved in emotional face processing^66^. We propose three possible explanations for the negative correlations between this pattern of neural activity when imitating emotional faces and social cognitive performance: 1) prolonged neural activity related to less efficient processing resulting in a greater magnitude of hemodynamic signal, as may be suggested by increased activity in regions associated with visual and face processing; 2) compensatory activity in which individuals with less efficient processing make greater use of the left IFG to compensate for lack of activity on the right (the compensatory hypothesis); or 3) over activity in the left IFG resulting in competition with right IFG, resulting in interference or an decrease in network coherence and deficits in social cognitive test performance (the dedifferentiation hypothesis). One possible explanation is that participants with poor performance may be attempting to compensate by activating aspects of the mentalizing system during lower-level social cognitive processing. It has been suggested that the anterior cingulate cortex, a region noted in the behavioral PLS, is involved in biasing activity towards either mentalizing or simulation^26^.

We did not find reliable relationships between the ER40 and RMET with neural activity to emotional faces. The ER40 and RMET rely on static images which fail to capture the full complexity of social cognitive processing, unlike the TASIT which requires interpretation of video scenarios of complex human interactions and the RAD which involves interpreting stories. It may be that more complex and process-general tests are required to more fully elicit relationships between activity in the simulation and mentalizing networks^26, 47^. Supporting this assertion, correlations between TASIT test scores and the observed voxel pattern in the PLS analysis were as high as 0.8, indicating a very strong relationship between mirroring network activity and social cognitive performance measured by TASIT1 (a lower-level task), as well as higher-level tasks TASIT2, and TASIT3. Some recent studies using more naturalistic higher-level social-cognitive paradigms, such as videos, have implicated IFG activity^25, 26^, and a small number of recent studies involving more complex social interaction tasks have noted co-activation in mirroring and mentalizing regions^24, 27, 38^.

Here we provide evidence that neural activation while imitating emotional faces is related to performance on dynamic and objective ‘real-life’ assessments of even the most complex social cognitive tasks. Given the relatively small sample size, further research expanding into a larger sample would be worthwhile to replicate and extend these findings. However, our findings suggest that patterns of neural activation during basic imitative behaviour may be a surrogate marker for highly complex social function in humans. The paradigm demonstrated here may be particularly useful for early identification studies in neurologic and psychiatric disorders with social cognitive impairment, and in intervention studies using ‘target engagement’ as a marker of early treatment response.

## Methods

### Participants

Twenty-eight healthy adult participants were initially included in this study. Average age of participants was 34.3 ± 11.7 years, with 18 men and 10 women, 23 right-handed participants, and an average level of education of 15.1 ± 2.22 years. Participants aged 18 to 55 were included in the study. All participants completed the Edinburgh handedness Inventory and were administered the Structured Clinical Interview for DSM-IV disorders (SCID) to rule out possible psychiatric illness. Urine toxicology screening was performed to further ensure that no participant with a current substance use disorder was included in the study. Additionally, exclusion criteria included a first degree relative with a history of psychotic mental disorder, a history of head trauma resulting in unconsciousness, or a history of seizure or other neurological disorders. The protocol was approved by the research ethics board of the Centre for Addiction and Mental Health, University of Toronto, all research was conducted in accordance with the declaration of Helsinki and the tri-council policy statement on Ethic Conduct for Research Involving Humans. All participants gave informed consent, and signed an institutionally approved informed consent form, prior to any research procedures.

### Social Cognitive Assessments

The Emotion Recognition (ER40) task consists of 40 images of whole faces making emotional expressions, with participants selecting from four possible emotional responses^34^. The Reading the Mind in the Eyes Test (RMET)^35^ consists of 36 trials showing only the eyes of a black and white face, with four possible choices to describe what the person is feeling (e.g. amused, irritated, cautious, contemplative). The RMET is considered a test of empathic abilities as it measures the ability to judge emotional states from looking at the eyes. The Relationships Across Domains (RAD) test^36^ presents 25 written vignettes of 2–4 lines followed by three statements which describe the behaviour of the male-female dyad from each vignette in domains of social life different from that vignette. Participants indicate if the behavior described in each statement is likely or unlikely to occur based on what was learned from the vignettes. The Awareness of Social Inference Test, Revised (TASIT) uses short video vignettes to measure emotional perception and theory of mind^37^. The TASIT is divided into three parts. Part 1 (TASIT1) consists of 24 short videos of actors portraying different emotional states (happy, sad, fear, disgust, surprise, and anger). Part 2 (TASIT2; social inference – minimal) consists of 15 videos showing sincere or sarcastic interactions between two actors, followed by four questions relating to what the actors were thinking, doing, meaning to say, and feeling. TASIT2 is considered a test of theory of mind. TASIT part 3 (TASIT3; social inference – enriched) consists of 15 vignettes in which the speaker is making an assertion which is literally untrue, but can represent either sarcasm or an attempt at deception. Success in TASIT3 requires the ability to detect deception in social encounters. In total, six social cognitive scores were derived from these tests (ER40 reaction time, RMET, RAD TASIT1, TASIT2, and TASIT3). Age was regressed out of all social cognitive test scores.

### MRI Scanning

MRI scanning was conducted on a Discovery 3 T MR750 machine from General Electric at the Centre for Addiction and Mental Health. The Imitate/Observe task was part of a longer multimodal MRI protocol to which each participant consented. Each block of the task (Imitate and Observe) was collected in counter-balanced order as a separate echo-planar imaging scan, with TRs = 3 sec, TE 30 ms, voxel size 3 mm isotropic, 50 slices, 64 × 64 matrix with FOV = 192 mm, flip angle = 77, and 110 TRs per scan. The first 5 TRs were excluded prior to any preprocessing to allow for magnetic steady-state.

### Imitate/Observe Task

The participants performed an imitate/observe task as previously described^13–15^ while being scanned. Participants were shown full-color photographs from an ethnically diverse set of 16 individuals (eight males and eight females) expressing five different facial expressions (fearful, sad, happy, angry, or neutral). During one scanning session, participants were instructed to imitate the expression on the faces (the *Imitate* session), while in the other session participants were instructed to observe the face (the *Observe* session). Each run consisted of 80 faces (16 per facial expression) plus 16 fixation trials presented in a pseudorandomized order determined using Optimize Design 11^67^ to maximize contrast efficiency. Each trial lasted three seconds, with the faces presented for two seconds and a jittered ISI (500 ms to 1500 ms). The order of sessions (Imitate or Observe) was counter-balanced across participants. Participants practiced the task prior to MRI scanning and were instructed to minimize motion during the imitation, only using their facial muscles when matching the expressions. A video recording during the scan confirmed that all participants were following instructions (i.e. imitating during the imitate session but not during observe).

### fMRI data analysis - Preprocessing

The initial preprocessing stages were slice time corrected and motion corrected in SPM8 (Wellcome Department of Cognitive Neurology, London, UK). Individual sessions were subjected to single-session independent components analysis (ICA, from the MELODIC module in FSL 5.0.6). ICA reports were visually examined, and any ICAs which were clear artifacts were removed based on a set of established criteria. On a case by case basis where some large motions contaminated the ICAs, data ‘scrubbing’ was performed using a spline interpolation. Generally, two TRs were removed for each motion spike, and no more than three TRs were permitted per motion spike and a maximum of three motion spikes were removed from any scan. Of the 56 sessions (Observe and Imitate for the 28 participants) scrubbing was performed in 16 sessions. In nine of these cases (four Observe sessions and five Imitate sessions) scrubbing was not successful. Possible reasons for unsuccessful ‘data scrubbing’ include large movements exceeding three TRs, more than three large movements, or over 90% of ICA components classified as artifact after data scrubbing. These sessions were subsequently excluded from further analysis, leaving neuroimaging data from 20 of the original 28 individuals for final analysis. Data were then de-noised based on the selected noise ICA components using FSL 5.0.6 (reg_filt). Normalization into MNI space was done using SPM8, and images were smoothed with an 8 mm Gaussian kernel.

### Partial Least Squares Analysis

Patterns of task related activity and relationships between social cognitive test scores and BOLD signal were examined using spatio-temporal PLS^31, 32^, a multivariate approach which allows for detection of spatial patterns and dependant variables across the brain without the need for a-priori selected contrasts across experimental conditions. PLS is designed to handle data with high collinearity while capturing essential non-redundant relationships among the data^31^, overcoming the confounding influence of collinear variables (e.g. cognitive tests) in traditional multiple regression. Additionally, PLS is also well suited to studying the relationships between numerous variables even in the presence of a relatively small sample, which is ideal for our purposes as it allows us to examine a range of social cognitive batteries. PLS produces latent variables (**LVs**) relating patterns of experiment task activity or behavioral measures with spatial patterns of neural activity across time points (scans, referred to as ***lags***, normalized to the scan in which the stimuli was presented, labeled as lag0). As a model free non-parametric approach, PLS is ideal to examine complex relationships amongst the battery of social cognitive scores and neural activity when imitating and observing emotional faces.

A task-PLS analysis was conducted to replicate the regions of activity in the SPM8 GLM. Brain data from an 18 second window (corresponding to 6 lags) was normalized to the first lag (lag0) to create a data matrix for each condition, stacked across participants. Cross covariance was calculated between the data matrix and a design matrix consisting of vector of experimental conditions (in the task PLS, emotional faces, neutral faces, and fixation crosses, separately for Imitate and Observe). The resulting cross-covariance matrix was then decomposed using singular value decomposition, which created a set of orthogonal latent variables (**LVs**) which optimally represent spatio-temporal relationships between voxels and experimental conditions. For each LV, a pattern of voxels at each lag value (the ‘brain pattern’) demonstrates the relationship with activity in the voxel at that lag and the ‘design pattern’ (representing the weights of each experimental event). Voxel weight is expressed as salience, which is proportional to the covariance of activity in that voxel and the design pattern expressed by that LV.

Behavioral PLS was used to examine relationships between social cognition and neural activity. Behavioral PLS examines patterns of covariance between scores and neural activity for each trial type across lags. As such the design pattern is the correlation between the overall pattern of neural activity in each condition and each social cognitive score. For the behavioral PLS, neural activity to emotional faces, neutral faces, and fixation (separately for imitate and observe) was related to the six social cognitive test scores from our battery, deterministically producing 36 LVs.

Statistical evaluation of each LV was performed using split-half permutation testing. A total of 500 permutations were run, with 100 split-half permutations within each. The overall permutation score determines if the effect represented by the LV is sufficiently strong to be differentiated from random noise, while the split-half analysis provides a measure of the stability of relationships between voxel patterns and design patterns in the data for each latent variable. As the permutations are performed at the level of the entire PLS analysis (rather than on individual LVs), multiple comparisons for the number of LVs is not necessary. In the split-half, the ‘BrainCorr’ value tests the reliability of the voxel pattern for a given design pattern associated with an LV, while ‘DesignCorr’ tests the reliability of a given voxel pattern for the behavioral pattern associated with that LV. Results are expressed as p-values. LV’s were considered significant if the overall permutation and at least one of the BrainCorr or DesignCorr was less than 0.05. A bootstrapping procedure with 1000 iterations was used to test if specific voxels were reliably related to the LV. A bootstrap ratio for each voxel was calculated as the voxel salience divided by its bootstrap standard error. A bootstrap ratio of 2.5 (corresponding to >95% reliability) was used to threshold all voxel pattern maps in PLS.

## References

[1] Mancuso F, Horan WP, Kern RS, Green MF (2011). Social cognition in psychosis: multidimensional structure, clinical correlates, and relationship with functional outcome. Schizophrenia research.

[2] Van Overwalle F, Baetens K (2009). Understanding others’ actions and goals by mirror and mentalizing systems: a meta-analysis. NeuroImage.

[3] Herbet G (2014). Inferring a dual-stream model of mentalizing from associative white matter fibres disconnection. Brain: a journal of neurology.

[4] Shamay-Tsoory SG, Aharon-Peretz J, Perry D (2009). Two systems for empathy: a double dissociation between emotional and cognitive empathy in inferior frontal gyrus versus ventromedial prefrontal lesions. Brain: a journal of neurology.

[5] Caspers S, Zilles K, Laird AR, Eickhoff SB (2010). ALE meta-analysis of action observation and imitation in the human brain. NeuroImage.

[6] Coricelli G (2005). Two-levels of mental states attribution: from automaticity to voluntariness. Neuropsychologia.

[7] Spunt RP, Lieberman MD (2013). The busy social brain: evidence for automaticity and control in the neural systems supporting social cognition and action understanding. Psychological science.

[8] Spunt RP, Lieberman MD (2012). Dissociating modality-specific and supramodal neural systems for action understanding. The Journal of neuroscience: the official journal of the Society for Neuroscience.

[9] Tunik E, Rice NJ, Hamilton A, Grafton ST (2007). Beyond grasping: representation of action in human anterior intraparietal sulcus. NeuroImage.

[10] Gallese V, Goldman A (1998). Mirror neurons and the simulation theory of mind-reading. Trends in cognitive sciences.

[11] Eisenberg N (2000). Emotion, regulation, and moral development. Annual review of psychology.

[12] Tangney JP, Stuewig J, Mashek DJ (2007). Moral emotions and moral behavior. Annual review of psychology.

[13] Dapretto M (2006). Understanding emotions in others: mirror neuron dysfunction in children with autism spectrum disorders. Nature neuroscience.

[14] Carr L, Iacoboni M, Dubeau MC, Mazziotta JC, Lenzi GL (2003). Neural mechanisms of empathy in humans: a relay from neural systems for imitation to limbic areas. Proceedings of the National Academy of Sciences of the United States of America.

[15] Pfeifer JH, Iacoboni M, Mazziotta JC, Dapretto M (2008). Mirroring others’ emotions relates to empathy and interpersonal competence in children. NeuroImage.

[16] Vogeley K (2001). Mind reading: neural mechanisms of theory of mind and self-perspective. NeuroImage.

[17] Samson D, Apperly IA, Kathirgamanathan U, Humphreys GW (2005). Seeing it my way: a case of a selective deficit in inhibiting self-perspective. Brain: a journal of neurology.

[18] Voineskos AN (2013). Neuroimaging evidence for the deficit subtype of schizophrenia. JAMA psychiatry.

[19] Wheeler AL (2014). Disrupted prefrontal interhemispheric structural coupling in schizophrenia related to working memory performance. Schizophrenia bulletin.

[20] Bliksted V, Fagerlund B, Weed E, Frith C, Videbech P (2014). Social cognition and neurocognitive deficits in first-episode schizophrenia. Schizophrenia research.

[21] Mikulan EP, Reynaldo L, Ibanez A (2014). Homuncular mirrors: misunderstanding causality in embodied cognition. Frontiers in human neuroscience.

[22] Caramazza A, Anzellotti S, Strnad L, Lingnau A (2014). Embodied cognition and mirror neurons: a critical assessment. Annual review of neuroscience.

[23] Corradini A, Antonietti A (2013). Mirror neurons and their function in cognitively understood empathy. Consciousness and cognition.

[24] Ciaramidaro A, Becchio C, Colle L, Bara BG, Walter H (2014). Do you mean me? Communicative intentions recruit the mirror and the mentalizing system. Social cognitive and affective neuroscience.

[25] Wolf I, Dziobek I, Heekeren HR (2010). Neural correlates of social cognition in naturalistic settings: a model-free analysis approach. NeuroImage.

[26] Zaki J, Hennigan K, Weber J, Ochsner KN (2010). Social cognitive conflict resolution: contributions of domain-general and domain-specific neural systems. The Journal of neuroscience: the official journal of the Society for Neuroscience.

[27] Zaki J, Weber J, Bolger N, Ochsner K (2009). The neural bases of empathic accuracy. Proceedings of the National Academy of Sciences of the United States of America.

[28] Marsh LE, Hamilton AF (2011). Dissociation of mirroring and mentalising systems in autism. NeuroImage.

[29] Marsh LE, Mullett TL, Ropar D, Hamilton AF (2014). Responses to irrational actions in action observation and mentalising networks of the human brain. NeuroImage.

[30] de Lange FP, Spronk M, Willems RM, Toni I, Bekkering H (2008). Complementary systems for understanding action intentions. Current biology: CB.

[31] McIntosh AR, Lobaugh NJ (2004). Partial least squares analysis of neuroimaging data: applications and advances. NeuroImage.

[32] Krishnan A, Williams LJ, McIntosh AR, Abdi H (2011). Partial Least Squares (PLS) methods for neuroimaging: a tutorial and review. NeuroImage.

[33] Wold S, Ruhe A, Wold H, Dunn IWJ (1984). The collinearity problem in linear regression. The partial least squares (PLS) approach to generalized inverses. SIAM Journal on Scientific and Statistical Computing.

[34] Kohler CG, Bilker W, Hagendoorn M, Gur RE, Gur RC (2000). Emotion recognition deficit in schizophrenia: association with symptomatology and cognition. Biological psychiatry.

[35] Baron-Cohen S, Wheelwright S, Hill J, Raste Y, Plumb I (2001). The “Reading the Mind in the Eyes” Test revised version: a study with normal adults, and adults with Asperger syndrome or high-functioning autism. Journal of child psychology and psychiatry, and allied disciplines.

[36] Sergi MJ (2009). Development of a measure of relationship perception in schizophrenia. Psychiatry research.

[37] McDonald, S., Flanagan, S. & Rollins, J. *The Awareness of Social Inference Test - Revised* (*TASIT-R*) (Pearson Assessment, 2011).

[38] Trapp K (2014). Imagining triadic interactions simultaneously activates mirror and mentalizing systems. NeuroImage.

[39] Spengler S, Bird G, Brass M (2010). Hyperimitation of actions is related to reduced understanding of others’ minds in autism spectrum conditions. Biological psychiatry.

[40] Brass M, Ruby P, Spengler S (2009). Inhibition of imitative behaviour and social cognition. Philosophical transactions of the Royal Society of London. Series B, Biological sciences.

[41] Rizzolatti G, Sinigaglia C (2008). Further reflections on how we interpret the actions of others. Nature.

[42] Casartelli L, Molteni M (2014). Where there is a goal, there is a way: what, why and how the parieto-frontal mirror network can mediate imitative behaviours. Neuroscience and biobehavioral reviews.

[43] de Bruin L, Gallagher S (2012). Embodied simulation, an unproductive explanation: comment on Gallese and Sinigaglia. Trends in cognitive sciences.

[44] Gallese V, Sinigaglia C (2011). What is so special about embodied simulation?. Trends in cognitive sciences.

[45] Agnew ZK, Bhakoo KK, Puri BK (2007). The human mirror system: a motor resonance theory of mind-reading. Brain research reviews.

[46] Van Overwalle F (2009). Social cognition and the brain: a meta-analysis. Human brain mapping.

[47] Zaki J, Ochsner KN (2012). The neuroscience of empathy: progress, pitfalls and promise. Nature neuroscience.

[48] Ochsner KN (2008). The social-emotional processing stream: five core constructs and their translational potential for schizophrenia and beyond. Biological psychiatry.

[49] Lisofsky N, Kazzer P, Heekeren HR, Prehn K (2014). Investigating socio-cognitive processes in deception: a quantitative meta-analysis of neuroimaging studies. Neuropsychologia.

[50] Iacoboni M, Dapretto M (2006). The mirror neuron system and the consequences of its dysfunction. Nature reviews. Neuroscience.

[51] Yang DY, Rosenblau G, Keifer C, Pelphrey KA (2015). An integrative neural model of social perception, action observation, and theory of mind. Neuroscience and biobehavioral reviews.

[52] Nelissen K (2011). Action observation circuits in the macaque monkey cortex. The Journal of neuroscience: the official journal of the Society for Neuroscience.

[53] Ishai A (2008). Let’s face it: it’s a cortical network. NeuroImage.

[54] Ishai A, Schmidt CF, Boesiger P (2005). Face perception is mediated by a distributed cortical network. Brain research bulletin.

[55] Bonda E, Petrides M, Ostry D, Evans A (1996). Specific involvement of human parietal systems and the amygdala in the perception of biological motion. The Journal of neuroscience: the official journal of the Society for Neuroscience.

[56] Grossman E (2000). Brain areas involved in perception of biological motion. Journal of cognitive neuroscience.

[57] Redcay E (2008). The superior temporal sulcus performs a common function for social and speech perception: implications for the emergence of autism. Neuroscience and biobehavioral reviews.

[58] Zilbovicius M (2006). Autism, the superior temporal sulcus and social perception. Trends in neurosciences.

[59] Kana RK, Libero LE, Hu CP, Deshpande HD, Colburn JS (2014). Functional brain networks and white matter underlying theory-of-mind in autism. Social cognitive and affective neuroscience.

[60] Lombardo MV (2010). Shared neural circuits for mentalizing about the self and others. Journal of cognitive neuroscience.

[61] Sperduti M, Guionnet S, Fossati P, Nadel J (2014). Mirror Neuron System and Mentalizing System connect during online social interaction. Cognitive processing.

[62] Spunt RP, Lieberman MD (2012). An integrative model of the neural systems supporting the comprehension of observed emotional behavior. NeuroImage.

[63] Libero LE (2014). The role of mirroring and mentalizing networks in mediating action intentions in autism. Molecular autism.

[64] Horan WP (2014). Self-reported empathy and neural activity during action imitation and observation in schizophrenia. NeuroImage. Clinical.

[65] Fishman I, Keown CL, Lincoln AJ, Pineda JA, Muller RA (2014). Atypical cross talk between mentalizing and mirror neuron networks in autism spectrum disorder. JAMA psychiatry.

[66] van Asselen M (2012). Scanning Patterns of Faces do not Explain Impaired Emotion Recognition in Huntington Disease: Evidence for a High Level Mechanism. Frontiers in psychology.

[67] Wager TD, Nichols TE (2003). Optimization of experimental design in fMRI: a general framework using a genetic algorithm. NeuroImage.

